# Heritability of semantic verbal fluency task using time-interval analysis

**DOI:** 10.1371/journal.pone.0217814

**Published:** 2019-06-11

**Authors:** T. P. Taporoski, N. E. Duarte, S. Pompéia, A. Sterr, L. M. Gómez, R. O. Alvim, A. R. V. R. Horimoto, J. E. Krieger, H. Vallada, A. C. Pereira, M. von Schantz, A. B. Negrão

**Affiliations:** 1 Institute of Psychiatry (LIM-23), Faculdade de Medicina FMUSP, Universidade de Sao Paulo, Sao Paulo, SP, Brazil; 2 Department of Biochemical Sciences, Faculty of Health and Medical Sciences, University of Surrey, Guildford, Surrey, United Kingdom; 3 Laboratory of Genetics and Molecular Cardiology, Heart Institute, Faculty of Medicine, University of São Paulo, São Paulo, SP, Brazil; 4 Departmento de Matemáticas, Universidad Nacional de Colombia, Manizales, Colombia; 5 Department of Psychobiology, Universidade Federal de São Paulo–Escola Paulista de Medicina, São Paulo, SP, Brazil; 6 Department of Psychological Sciences, University of Surrey, Guildford, Surrey, United Kingdom; 7 Postgraduate Program in Public Health, Federal University of Espírito Santo, Vitória, ES, Brazil; University of California San Diego, UNITED STATES

## Abstract

Individual variability in word generation is a product of genetic and environmental influences. The genetic effects on semantic verbal fluency were estimated in 1,735 participants from the Brazilian Baependi Heart Study. The numbers of exemplars produced in 60 s were broken down into time quartiles because of the involvement of different cognitive processes—predominantly automatic at the beginning, controlled/executive at the end. Heritability in the unadjusted model for the 60-s measure was 0.32. The best-fit model contained age, sex, years of schooling, and time of day as covariates, giving a heritability of 0.21. Schooling had the highest moderating effect. The highest heritability (0.17) was observed in the first quartile, decreasing to 0.09, 0.12, and 0.0003 in the following ones. Heritability for average production starting point (intercept) was 0.18, indicating genetic influences for automatic cognitive processes. Production decay (slope), indicative of controlled processes, was not significant. The genetic influence on different quartiles of the semantic verbal fluency test could potentially be exploited in clinical practice and genome-wide association studies.

## Introduction

The historical roots of the semantic verbal fluency (SVF) task lie in tests requiring the production of words from a certain semantic category [1]. This task involves long-term memory retrieval according to the meaning of words, or semantic memories [2]. The most common category used in SVF tests is “animals”, a semantic category with minimal differences across countries, generations, and feasible across different education levels [3]. Nonetheless, previous SVF studies have demonstrated that demographic characteristics such as sex [4], age [5], time of day [6], and education [7, 8] can influence performance. The task is broadly used in clinical and non-clinical research [9], mainly because its sensitivity to several neurobiological conditions associated with cognitive impairment, such as Alzheimer’s disease [10], ageing [11], mild cognitive impairment [12], schizophrenia [13], and Parkinson’s disease [14] among others.

Despite the simplicity of the SVF task, the array of cognitive abilities underlying performance is large, including hierarchically organized semantic storage and retrieval, working memory, and/or executive functions, speed of lexical access, and other domains that reflect a person’s general verbal functioning [15, 16]. Moreover, the frequency of words produced falls progressively throughout the task, and the cognitive domains used are assumed to vary accordingly. While the production of the first words are thought to primarily involve automatic retrieval from semantic networks, and/or access to the semantic knowledge network [15], subsequent performance demands an increase of cognitive effort as the task progresses due to exhaustion of animal subcategories and lower frequency of target words [17, 18]. This effort involves monitoring and suppression of exemplars to avoid repetitions, as well as strategic generation of cues to access new words [19]. Thus, towards the end the task becomes increasingly difficult, and more dependent on executive abilities [20].

Twin- and family-based methods allow the estimation of genetic contributions to individual differences in cognitive traits such as those involved in the SVF test. Heritability represents the proportion of the phenotypic variance attributable to genetic effects, and can vary between zero and one, with higher values indicating stronger genetic effects [21]. Published family and twin-based studies have provided heritability estimates varying from 26% to 85% for general verbal abilities such as vocabulary, semantic fluency, phonemic fluency and reading [22]. Heritability of total SVF scores has been examined previously demonstrating that the variance for this phenotype is neither explained by genetic nor by environmental factors alone [23]. Four twin studies showed a heritability varying from 20% to 54% [24–27]. Furthermore, four family-based studies estimated heritabilities between 32% and 52% [28–31]. All these studies only considered performance in the total number of generated exemplars during 60 s. However, it should be considered that each specific cognitive domain tends to respond with a diverse proportion to genetic underpinnings. The variation in heritability estimates of cognitive domains can reflect the nature of the trait, distribution of the phenotype along that particular population and methodological approach, control of different confounders, as well as the characteristics of each sample [32]. Only one of these prior studies had a population comprised of extended families [30]. This suggests a need for more studies using this approach to extend knowledge on heritability of SVF performance. It could also be argued that because different cognitive domains are involved at different stages of the SVF, heritability and environmental factors could differ as the task progresses, and that this could have confounded data from prior studies in which this was not taken into account. The extended family paradigm was used in the present study, in which we examined SVF across the entire 60-minute interval with data from an extended family cohort, the Baependi Heart Study. We also examined heritability and environmental effects on the production across time quartiles of a total of 60 s, which has not been previously explored.

The town of Baependi is located in the southeast of Brazil, and presents low rates of migration and cultural disparity. Unlike most published studies investigating the heritability of SVF, which were performed in participants of entirely European descent, the Baependi cohort is highly admixed between European, African, and Native American ancestries [33]. Furthermore, it includes wide ranges of age, socio-economic status and (in contrast to the majority of studies that investigate cognition) a particularly wide variety of educational backgrounds, ranging from illiterate individuals to post-graduate degree holders. Therefore, the diverse sociodemographic characteristics of the sample provide a robust way to exploit the relationship of genetics and environmental effects on cognitive domains.

We aimed to explore the variation in the proportion of genetic, demographic and environmental factors involved in performance in the SVF task over 60 s and also in four-time quartiles of 15 s [0-15s (T1), 16-30s (T2), 31-45s (T3), 46-60s (T4)] [34]. We hypothesised that the known involvement of different cognitive domains across the sampling periods would be reflected in variations in heritability of performance in different time quartiles.

## Materials and methods

### Participants

Data were collected from 1,735 participants belonging to 134 extended families within the Baependi Heart Study. The study protocol was approved by the Ethics committee of the Hospital das Clínicas, University of São Paulo, Brazil, and complied with international ethical standards on human experimentation. The methodology for recruitment has been described previously [35]. Briefly, all extended family members aged above 18 years of probands who were randomly selected from 11 out of 12 census districts in Baependi were invited to participate. All participants provided written informed consent. For illiterate individuals, the researcher read the content aloud and collected the authorization of a legal representative in agreement with the participant's decision to participate in the study. The two examiners (one of whom was the first author) were trained psychologists under supervision during data collection. Only one participant was excluded from the study because he was unable to speak as a consequence of a stroke.

### Instruments

Data were collected between April 2013 and March 2016 in the permanent research station of the study, based in the centre of Baependi. The assessment was made using the SVF task. Participants were asked to orally name as many animals as possible within 60 s, avoiding repetitions and variations of the same animal. Errors were considered repetitions of the same animal, higher order taxonomic categories when lower order units were present and variations according to gender of exemplars (e.g., *hen* and *rooster*) or age (e.g., *dog* and *puppy*). The first analysis was based on the total score (number of animal exemplars produced in 60 s). In order to better comprehend the contributions of the genetic and covariates in performance in the task that reflect automatic and controlled processes as explained above, examiners partitioned the answers into four 15-s quartile bins.

### Statistical analysis

The results reflect the values for the full sample with the exclusion of two participants who were found to be 3 standard deviation above or below the mean, whose data created distortions in the statistical analysis. Descriptive statistics were estimated using R software environment (http://cran.r-project.org/). When the normality assumption did not hold for a specific trait, log-transformation was applied followed by a new data assessment. All variables reached distribution estimates within the normal range according to skewness (60 s = 0.45, T1 = 0.70, T2 = 0.51) and kurtosis (60 s = 2.98, T1 = 3.66, T2 = 3.49) test with the exception of the scores in T3 and T4, which were *log*-*transformed* (T3 = log base 6, T4 = log base 7) to satisfy parametric test assumptions and reached the following test measures: skewness (T3 = -0.25, T4 = -0.35) and kurtosis (T3 = 3.02, T4 = 2.60). However, in order to enable the reader to compare the effects at each quartile, beta values were kept in the same scale as non-transformed values.

All response variables were analysed as continuous, and polygenic heritability estimates were calculated from polygenic mixed models (or variance component approach), implemented in the SOLAR package [36].

The variance component model is a well-established tool for heritability estimates in family studies [21, 37–39] and briefly described in the following. The model decomposed the overall variance of the phenotype into its genetic and environmental sources using the maximum likelihood methods given the covariance among family members. In this method, the likelihood ratio test (LRT) is applied to test whether the additive polygenic effect in each analysis is accounted for, by a significant component of the variation for the trait under study, after adjusting for the covariates. In the model, the heritability estimate represents the proportion of the phenotypic variance attributable to addictive genetic effects and is given by h^2^ = σ^2^g/σ^2^p, where σ^2^g is the variance due to the addictive effects of genes, and σ^2^p is the phenotypic variance, where both were already adjusted for the covariate effects.

Under the assumptions of the variance components model (or polygenic mixed model), we performed heritability calculations of the continuous variables using both unadjusted and adjusted models for covariates. Specifically for the present study, total score over 60 s, and 15-s quartiles were controlled for covariates, and the best-fitting model according to a stepwise approach included sex, age, schooling, and time of day as covariates. We recorded age (18–91), schooling (0–23 years), and time of day when the task was carried out (7–20, representing 0700h-2000h). The stepwise approach consisted in adding the covariates to the model one by one. Consequently, all covariates that reached the level of statistical significance were placed together in the same model, and the ones that remained significant in the presence of other covariates formed part of the best-fit model. Socioeconomic status, depression symptoms, chronotype/diurnal preference, and interaction effect between age and schooling were also tested as covariates but were not significant. For this reason, age and schooling were analysed separately as individual effects.

As a last supplemental analysis, we ran a linear regression using the total number of words (min = 3, max = 32) explained by the production of each one of the four time intervals (T1, T2, T3, T4). From this analysis, we extracted the ‘intercept’, describing the sample’s average starting point (automatic processes), and the ‘slope’, representing the decay that occurs over time (increased use of controlled processes towards the end of the task) [40]. We then estimated heritability for the two parameters, as the response variables, and adjusted for the best-fit model. This method has been used in genetic and cognitive studies before, and consists of a two-step modelling which firstly reduces repeated measures to one summary statistics followed by a standard genetic analysis in which covariates were controlled for.

## Results

### Sample characteristics

Sociodemographic characteristics and results of the SVF task (total score for 60 s) across gender, age and schooling are shown in Table 1.

**Table 1:** Mean(±SD) of generated animal exemplars and errors (repetitions) during the total 60 s semantic verbal fluency task according to socio-demographic characteristics.

		Animals (±SD)	Errors (±SD)
**Total**	(n = 1735)	14.36 (4.66)	0.69 (0.94)
**Sex**	Male (n = 694)	14.38 (4.66)	0.61 (0.90)
	Female (n = 1041)	14.34 (4.67)	0.75 (0.97)
**Age**	18–27 (n = 252)	16.08 (4.37)	0.66 (0.91)
	28–37 (n = 320)	16.11 (4.82)	0.73 (0.99)
	38–47 (n = 318)	14.53 (4.62)	0.74 (0.97)
	48–57 (n = 385)	13.83 (4.32)	0.61 (0.91)
	58–67 (n = 278)	13.15 (4.36)	0.75 (0.89)
	68–77 (n = 122)	12.04 (4.10)	0.71 (1.06)
	>77 (n = 60)	10.62 (3.16)	0.70 (0.88)
**Schooling**	Illiterate (n = 106)	10.22 (3.40)	0.53 (0.82)
	≤ 4 years (n = 575)	12.18 (3.66)	0.64 (0.94)
	5–8 years (n = 304)	14.00 (4.33)	0.77 (1.04)
	9–11 years(n = 442)	15.81 (3.91)	0.70 (0.90)
	≥12 years (n = 287)	18.41 (4.56)	0.79 (0.93)

Note: age and schooling are presented in bins for descriptive purposes but were used as continuous variables in the analyses.

Results of the verbal fluency task for all time intervals across gender, age and schooling are shown in Table 2.

**Table 2:** Mean(±SD) number of exemplars in the semantic verbal fluency task in each of the four 15-s time quartiles (T1 to T4) according to socio-demographic characteristics.

		Animals(±SD)			
		T1 (0–15 s)	T2 (16–30 s)	T3 (31–45 s)	T4 (46–60 s)
**Total**		5.85 (1.94)	3.58 (1.72)	2.65 (1.58)	2.28(1.63)
**Sex**	Male	5.79 (1.98)	3.54 (1.67)	2.68 (1.58)	2.39 (1.66)
	Female	5.88 (1.91)	3.60 (1.76)	2.63 (1.57)	2.21 (1.60)
**Age**	18–27	6.74 (2.01)	3.96 (1.74)	2.86 (1.61)	2.52 (1.68)
	28–37	6.55 (1.94)	4.12 (1.88)	3.01 (1.62)	2.42 (1.64)
	38–47	5.92 (1.74)	3.65 (1.66)	2.71 (1.68)	2.25 (1.74)
	48–57	5.67 (1.80)	3.30 (1.62)	2.57 (1.52)	2.29 (1.61)
	58–67	5.26 (1.75)	3.37 (1.61)	2.47 (1.47)	2.05 (1.43)
	68–77	4.61 (1.62)	3.05 (1.56)	2.19 (1.31)	2.20 (1.67)
	>77	4.25 (1.43)	2.57 (1.24)	1.87 (1.37)	1.93 (1.47)
**Schooling**	Illiterate	4.30 (1.31)	2.54 (1.41)	1.70 (1.28)	1.68 (1.47)
	≤ 4 years	5.01 (1.48)	2.93 (1.42)	2.23 (1.38)	2.00 (1.48)
	5–8 years	5.85 (1.91)	3.40 (1.60)	2.64 (1.59)	2.10 (1.60)
	9–11 years	6.48 (1.84)	4.05 (1.62)	2.87 (1.49)	2.41 (1.60)
	≥12 years	7.13 (1.97)	4.71 (1.87)	3.52 (1.70)	3.06 (1.78)

Note: age and schooling are presented in bins for descriptive purposes but were used as continuous variables in the analyses.

Phenotypic correlation among traits was calculated both with Pearson and bivariate correlation provided by SOLAR, which corrects correlation estimates for family structure. As results were similar in the two methods, we showed only Pearson correlation given the higher facility in estimating p value and CI (95%) when using this method. Pearson correlations among quartiles were all significant (p<0.05) but low, and decrease when quartiles that were further apart in time were compared: T1 x T2 r = 0.36 (CI: 0.31–0.40); T1 x T3 r = 0.24 (CI: 0.20–0.29); T1 x T4 r = 0.17 (CI: 0.12–0.21); T2 x T3 r = 0.39 (CI: 0.35–0.43); T2 x T4 r = 0.25 (CI: 0.20–0.29); T3 x T4 r = 0.28 (CI: 0.24–0.36).

### Heritability estimates

Table 3 shows heritability estimates obtained by the variance component model for performance during the 60-s interval and the four 15-s quartiles. We considered four statistical models: No adjustment, Model I–adjustments to age and sex, Model II–adjustments to age, sex and schooling and, Model III–adjustments to age, sex, schooling and time of day.

**Table 3:** Random effect values—family heritability estimates on generated animal exemplars during the total 60 s of the semantic verbal fluency task and per 15-s time quartiles (T1-T4).

Model	Total (60 s)	T1 (0–15 s)	T2 (16–30 s)	T3 (31–45 s)	T4 (46–60 s)
**No adjustments**					
Heritability	0.32	0.22	0.21	0.23	0.06
h^2^ standard error	0.05	0.05	0.05	0.05	-
p value CI (95%)	<0.01 (0.30;0.34)	<0.01 (0.20;0.24)	<0.01 (0.19;0.23)	<0.01 (0.21;0.25)	0.06 -
**Model I–age, sex**					
Heritability	0.46	0.33	0.28	0.28	0.07
h^2^ standard error	0.05	0.05	0.05	0.05	0.04
p value CI (95%)	<0.01 (0.47;0.48)	<0.01 (0.31;0.35)	<0.01 (0.26;0.30)	<0.01 (0.26;0.30)	<0.01 (0.06;0.08)
**Model II–age, sex, schooling**					
Heritability	0.21	0.18	0.12	0.12	<0.01
h^2^ standard error	0.05	0.05	0.05	0.05	0.06
p value	<0.01	<0.01	<0.01	<0.01	<0.01
CI (95%)	(0.19;0.23)	(0.16;0.20)	(0.10;0.14)	(0.10;0.14)	-
**Model III–age, sex, schooling, time of day**					
Heritability	0.21	0.17	0.09	0.12	<0.01
h^2^ standard error	0.05	0.05	0.04	0.05	-
p value	<0.01	<0.01	<0.01	<0.01	0.50
CI (95%)	(0.20;0.21)	(0.16;0.17)	(0.08;0.09)	(0.11;0.12)	-

In the unadjusted model, heritability for the 60-s period was 0.32 and then increased (h^2^ = 0.46) when age and sex were added. The addition of schooling (model II) had the strongest influence on the heritability estimate leading to a sharp decrease (h^2^ = 0.21) in additive variance. Model III confirmed a better performance later in the day, but time of day did not change the heritability value obtained from Model II. The best-fit model (Model III h^2^ = 0.21) contained age, sex, schooling and time of day as covariates. In Model III heritability was highest at T1 (0.17) compared to the other time quartiles (T2 = 0.09, T3 = 0.12 and T4 = 0.0003). The variance component method was able to capture the source of variances of all time-interval excepted for T4 in the unadjusted model and Models II and III. S1 Fig in Supplemental Material presents a summary of mean score for animal exemplars and heritability estimates for the best-fit model.

### Fixed effect adjustments: Age, sex, schooling, and time of day

Results for the effect of age, sex, schooling, and time of day on word production are shown in Table 4.

**Table 4:** Fixed effect values–covariates influence to best-fit model of generated animal exemplars during the total 60 s of the semantic verbal fluency task and per 15-s time quartiles (T1-T4).

	Total (60 s)	T1 (0–15 s)	T2 (16–30 s)	T3 (31–45 s)	T4 (46–60 s)
**Schooling**					
β	0.478	0.152	0.142	0.108	0.090
p value	< 0.01	< 0.01	< 0.01	< 0.01	< 0.01
**Age**					
β	-0.021	-0.019	-0.002	<-0.01	0.003
p value	< 0.01	< 0.01	0.400	0.840	0.210
**Sex**					
β	-0.398	-0.029	-0.028	-0.111	-0.227
p value	0.039	0.720	0.720	0.130	< 0.01
**time of day**					
β	0.115	0.050	0.028	0.022	0.016
p value	< 0.01	< 0.01	< 0.01	0.025	0.130

Covariates coded as schooling. years of schooling from 0 to 23; sex. 1 for men and 2 for women; age. from 18 to 91; time of day. from 7 (representing 7.00 am) to 20 (representing 8.00 pm–when the last time of assessment took place).

Schooling was the only covariate that was significant for all time intervals. The magnitude of the schooling effect remained strong over the whole course of the task. For the total score, for example, two extra years of schooling elevated performance by almost one word on average. Age exerted a negative effect mainly in the very beginning of the task (T1), also visible in the 60-s total score. Each incremental decade of age decreased performance by 0.2 words. The magnitude of the sex effect was low but significant for the 60-s and T4 measures, with men achieving higher means of produced words of 0.40 and 0.23, respectively. Although time of day did not change the additive variance when compared to Model II, its influence can be seen during the whole task except at T4. The positive direction of the β value indicates better performance in later hours of assessment during the day.

Adjusted heritability results for the two parameters extracted from the linear regression model were as follows: The intercept showed a mean = 6.49, SD = 2.34, h^2^ = 0.18, SE = 0.05, p value< 0.01 with significant influence of age, schooling and time (all covariates p values< 0.01). For slope, results were: mean = -1.16, SD = 0.74, with a non-significant heritability estimate (h^2^ = 0.06, SE = 0.05, p value = 0.08), after controlling for the same covariates as above.

### Descriptive measures

Descriptive measures are displayed in Table 1. Categories were stratified into age decades and education landmarks to enable general descriptive comparisons. Means stratified by sex illustrate the similarity in performance in both categories, despite the significant differences favouring men for the entire 60-s and the T4 measures, confirming the mixed-model analysis. The lowest two age categories revealed comparable results in all time quartiles, followed by a constant decrease in production from the age bin of 40–50 years. Scores increased linearly as a function of schooling using scores for the 60 s and in all 15-s time-quartile measures. Errors by repetitions of animal names were minimal and were not analysed statistically. Production of words that were not animals only occurred in two individuals and is therefore not described here. S1 Table presents dispersion measures.

## Discussion

Performance in the SVF task in the Baependi sample was sensitive to demographic and environmental factors, as previously described in the literature from other locations [4, 5, 8]. In this study, unadjusted heritability estimates for 60-s score increased with the addition of sex and age, and decreased when years of schooling was considered as a covariate. Although assessments at later times of day correlated positively with performance as found before [6], they did not change heritability estimates. The best fitting heritability model included age, sex, schooling and the time of day as covariates, but the biggest impact on performance was schooling.

The heritability estimate for Model I (0.46), addressing 60-s word production, confirmed a genetic basis for SVF as reported in family and twin cohorts [22]. However, considering the best-fit model (Model III), our heritability estimates lay on the lower end compared to the majority of previous family studies [28–31, 41]. This can probably be attributed to the fact that, in our study, education was assessed as a continuous variable (years of schooling) within a very considerable variety of education levels, reflecting the influence of schooling more comprehensively than educational categories. Furthermore, some studies using this test were composed of more homogeneous and higher schooled samples than ours [29, 41], which can underpower the modulating effect of education and inflate the genetic contribution in the heritability estimate, as seen in our study before controlling for schooling. Indeed, education was the covariate with the biggest impact on the additive variance of verbal fluency performance since heritability diminished from 0.46 in Model I to 0.21 in Models II and III. The strength of its influence was significant and high in the final model. Thus, our results corroborate prior reports that formal education exerts an important effect on SVF performance, as found for other cognitive tasks [42]. As expected, while schooling was associated with better performance [8], we observed a negative influence of age on word production [43]. The observation of a decline in semantic fluency from the age of 40 is also in line with previous reports, supporting the non-linear effect of age on performance [5] which could be related to the speed of lexical access that can be modulated by age [44]. By contrast, the impact of sex on the task is somewhat inconsistent in the literature. Whereas some studies report women significantly outperforming men [4], others found no significant differences, with a slight advantage for men [7, 8]. As mentioned before, in our study, the small sex differences in performance were statistically relevant only for 60 s (p = 0.04) and T4 (p<0.01) and may have reached significance due to our large sample size.

In order to gain a better picture of the roles played by genetics and demographic/environmental variables during the task, we analysed performance by sampling of time-quartiles obtained during the 60-s duration of the task. In the analysis by quartiles, word production peaked at T1 and sharply declined from then onwards, which is in accordance with previous studies [12, 15, 45]. This pattern of response has been attributed to two underlying cognitive processes, automatic semantic retrieval and executive control [15, 16, 46]. It has been hypothesised that in the beginning of the task, word production relies heavily on automatic semantic retrieval processes, which allow access to many commonly used animal names [17]. Executive processes then become progressively important until the final part of the task due to monitoring to avoid repetitions and exhaustion of the pool of words, which also involves retrieval of words with lesser frequency of use and that are therefore harder to bring to mind [17]. Thus, controlled executive strategies to find new words and inhibit repetitions are needed to complete the task successfully. The time-quartile heritability estimate was at its highest at T1, which reflects the genetic components underlying possible automatic cognitive process. It has been suggested that between T1 and T2, the recruited cognitive abilities manifest in the task shifts from predominantly automatic to mainly controlled processes [12, 20, 45, 46]. In our data, the decline in heritability observed between T1 and T2 confirms that the cognitive abilities used at the start of the task are different from the ones used between 16 and 30 s, the former having been more sensitive to genetic variation when confounders are controlled for.

Phenotypic correlations among quartiles were all low and decrease when comparing quartiles that were further apart in time, indicating that there was a decrease in shared cognitive processes as the task progresses. As the number of generated animal exemplars decreased during the task, it is possible that heritability and correlation estimates could be influenced by the lower variance in the last quartiles. Performance amongst the quartiles of time are not totally independent because they were produced by the same person across a period of time. To address these issues, we performed a linear regression analysis, where the exemplar production across each one of the time-intervals was considered by the four equidistant time-points (15s), reducing scores to two measures [47]: intercept (the individual’s average starting point) and slope (changes on production over time). These individual metrics respectively describe the automatic and controlled processes involved in the task based on suggestions of prior studies that have been mentioned above. Hence, higher intercept values would represent a good performance at the beginning of the task, while lower slopes indicate better ability to sustain a constant production during the whole task. The best-fit heritability model controlled for significant confounders for the intercept (h^2^ = 0.18) was similar to that of the analysis per quartile for T1 (h^2^ = 0.17); for slope, heritability did not express a significant genetic variance (h^2^ = 0.06, p = 0.08), indicating a much stronger genetic influence for the purported automatic than controlled processes, even when the phenotype relatedness and covariates were considered. Heritability estimates from these parameters confirmed the main findings using other statistical technique: The intercept, expressing the number of animals named during T1 in relation to the decay in the following time-quartiles, kept its genetic weight, whilst the slope, expressing a single measure for exemplar production decay between T2, T3 and T4, did not express a significant genetic background.

Parallel to the progressive decline in the influence of genetic factors on word production as performance in the task, schooling maintained its major role in the variability for the trait at all time points. This can be due to participants’ vocabulary and executive functioning, which contribute to performance in the semantic fluency task and are positively influenced by education [48]. Thus, it may well be that the genetic contribution to performance at T2 and T3 is indeed more related to controlled processes, that are enhanced in higher educated individuals, than automatic ones. Our data from T4 adds little to the understanding of biological factors contributing to performance in SVF because Model III was not significant for this quartile. Taken together, we interpret heritability of the 60-s semantic fluency score to reflect a combined genetic influence of distinct cognitive processes. Partitioning the task into time quartiles made it possible to detect genetic influences during the task. These genetic influences occurred predominantly on processes during the first seconds of the task, which prior studies have shown to be due to automatic cognitive abilities [12, 20, 45, 46].

Accordingly, it seems justifiable in clinical settings, where a fast screening resulted from the total number of words is intended, to restrict semantic fluency tasks to 15 s as a proxy of 60 s production, as previously proposed, since: (a) 45% of the total 60 s score was produced during T1, and (b) performance in this quartile showed the highest underlying biological substrate in the form of higher heritability. Semantic fluency tasks that last less than 60 s may, therefore, be sufficient to describe factors that are known to influence SVF and might be more appropriate for genetic investigations such as genome-wide association studies targeting performance in SVF. This is so because this approach may enable researchers to disentangle possible contribution to performance during the usual 60 s task from distinct cognitive abilities that seem to have different genetic bases and show different susceptibility to environmental factors.

Taking into account that there is no previous study examining the heritability of SVF broken down by individual time quartiles, future investigations considering the genetic bases of 60 s performance partitioned into time-bins in family and twin studies, using the category animals or others (e.g., supermarket items, actions, etc.) would be an important additional contribution to this work. It would also be important to explore the heritability of automatic and controlled cognitive abilities captured in tests that have been proven to be sensitive to these cognitive domains, and compare their estimates with the one generated by the SVF four time quartiles. Heritability estimates controlled for the confounders described in the literature were intermediate or low, leading us to the conclusion that there must be other environmental factors that determine interindividual variability and which are not being estimated, but should be taken into further consideration. One of these factor that has recently been shown to have an important role on cognitive performance is neurotoxic pollution [49] and it would be interesting to consider it as a potential significant covariate in future studies.

In the present study, no participants were excluded based on cognitive limitations or diseases, which can be regarded as a limitation as this introduces confounders into the analysis. However, we consider this a potential advantage, since the sample reflects the normal phenotypic variance in the community. Moreover, some of the categories into which the analyses were stratified are discrepant in terms of representativity (e.g., women, the middle-aged, and intermediate levels of schooling were present in larger numbers), and could have represented a source of bias. Notwithstanding, we believe this did not impact our findings, as statistical precautions were taken to adjust the analysis according to these discrepancies and the sample was large.

## Supporting information

S1 Fig Combining performance and genetic components along the semantic verbal fluency task. Best-fit model summary of the mean scores and heritability estimates for 60 s and all time intervals.
(DOCX)

S1 Table Variance measures for 60 seconds and all time intervals of semantic verbal fluency task. Central and dispersion measures for semantic fluency production during 60 seconds and the four quartiles of time.
(DOCX)
