# Supplementary material for: Heritability of semantic verbal fluency task using time-interval analysis
Source: PLoS One. 2019 Jun 11;14(6):e0217814. doi: 10.1371/journal.pone.0217814 (PMC6559646; doi:10.1371/journal.pone.0217814)
Supplement: S1 Fig — Best-fit model summary of the mean scores and heritability estimates for 60 s and all time intervals. (DOCX) [file pone.0217814.s001.docx]

**S1 Figure. Combining performance and genetic components along the semantic verbal fluency task**


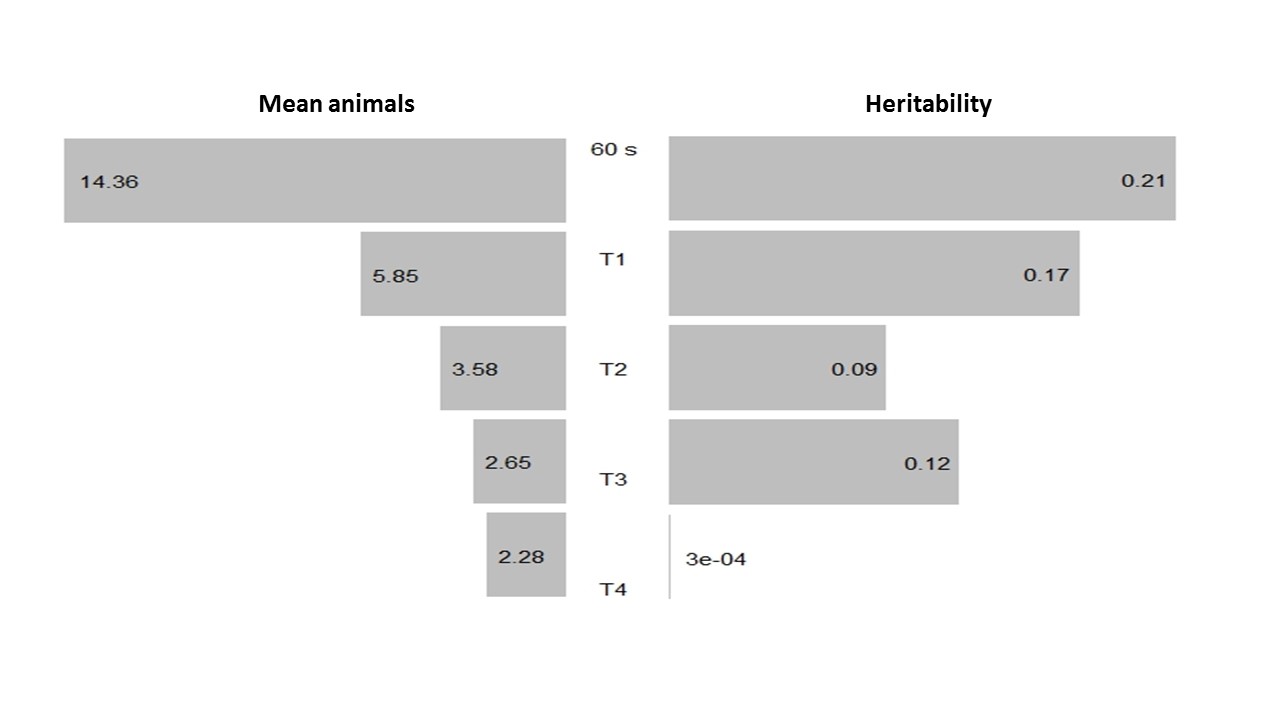


Best-fit model summary of the mean scores and heritability estimates for 60 s and all time intervals
