# Supplementary material for: Heritability of semantic verbal fluency task using time-interval analysis
Source: PLoS One. 2019 Jun 11;14(6):e0217814. doi: 10.1371/journal.pone.0217814 (PMC6559646; doi:10.1371/journal.pone.0217814)
Supplement: S1 Table — Central and dispersion measures for semantic fluency production during 60 seconds and the four quartiles of time. (DOCX) [file pone.0217814.s002.docx]

**S1 Table.** **Variance measures for 60 seconds and all time intervals of semantic verbal fluency task**

|  |  | **Total**  (60s) | **T1**  (0-15s) | **T2**  (16-30s) | **T3**  (31-45s) | **T4**  (46-60s) |
| --- | --- | --- | --- | --- | --- | --- |
|  | **mean** | 14.36 | 5.85 | 3.58 | 2.65 | 2.28 |
|  | **standard deviation** | 4.66 | 1.94 | 1.72 | 1.58 | 1.63 |
|  | **median** | 14 | 6 | 3 | 3 | 2 |
|  | **variance** | 21.77 | 3.76 | 2.97 | 2.49 | 2.66 |
|  | **variance coefficient** | 32.49% | 33.15% | 48.15% | 59.56% | 71.44% |
|  | **minimum** | 4 | 1 | 0 | 0 | 0 |
|  | **maximum** | 32 | 14 | 11 | 11 | 10 |

Central and dispersion measures for semantic fluency production during 60 seconds and the four quartiles of time.
